# Disrupted gut microecology after high-dose ^131^I therapy and radioprotective effects of arachidonic acid supplementation

**DOI:** 10.1007/s00259-024-06688-9

**Published:** 2024-04-02

**Authors:** Ganghua Lu, Dingwei Gao, Wen Jiang, Xiaqing Yu, Junyu Tong, Xiaoyan Liu, Tingting Qiao, Ru Wang, Mengyu Zhang, Shaoping Wang, Jianshe Yang, Dan Li, Zhongwei Lv

**Affiliations:** 1 Clinical Nuclear Medicine Center, Imaging Clinical Medical Center, Institute of Nuclear Medicine, Department of Nuclear Medicine, Shanghai Tenth People’s Hospital, School of Medicine, Tongji University, Shanghai, 200072 China; 2 Department of Nuclear Medicine, Sun Yat-Sen Memorial Hospital, Sun Yat-Sen University, Guangzhou, 510289 China

**Keywords:** ^131^I therapy, Gut microbiota, Fecal metabolites, Arachidonic acid, Hydroxy-3-methylglutaryl-coenzyme A synthase 1

## Abstract

**Background:**

Despite the potential radiotoxicity in differentiated thyroid cancer (DTC) patients with high-dose ^131^I therapy, the alterations and regulatory mechanisms dependent on intestinal microecology remain poorly understood. We aimed to identify the characteristics of the gut microbiota and metabolites in DTC patients suffering from high-dose ^131^I therapy and explore the radioprotective mechanisms underlying arachidonic acid (ARA) treatment.

**Methods:**

A total of 102 patients with DTC were recruited, with fecal samples collected before and after ^131^I therapy for microbiome and untargeted and targeted metabolomic analyses. Mice were exposed to total body irradiation with ARA replenishment and antibiotic pretreatment and were subjected to metagenomic, metabolomic, and proteomic analyses.

**Results:**

^131^I therapy significantly changed the structure of gut microbiota and metabolite composition in patients with DTC. *Lachnospiraceae* were the most dominant bacteria after ^131^I treatment, and metabolites with decreased levels and pathways related to ARA and linoleic acid were observed. In an irradiation mouse model, ARA supplementation not only improved quality of life and recovered hematopoietic and gastrointestinal systems but also ameliorated oxidative stress and inflammation and preserved enteric microecology composition. Additionally, antibiotic intervention eliminated the radioprotective effects of ARA. Proteomic analysis and ursolic acid pretreatment showed that ARA therapy greatly influenced intestinal lipid metabolism in mice subjected to irradiation by upregulating the expression of hydroxy-3-methylglutaryl-coenzyme A synthase 1.

**Conclusion:**

These findings highlight that ARA, as a key metabolite, substantially contributes to radioprotection. Our study provides novel insights into the pivotal role that the microbiota-metabolite axis plays in radionuclide protection and offers effective biological targets for treating radiation-induced adverse effects.

**Supplementary Information:**

The online version contains supplementary material available at 10.1007/s00259-024-06688-9.

## Introduction

^131^I has been widely used in the treatment of differentiated thyroid cancer (DTC) since the 1940s, and most patients treated with a therapy dose of 30–100 mCi do not have significant radiation toxicity after primary ^131^I therapy [1, 2]. Unfortunately, some patients with metastases or recurrence of thyroid carcinomas may require multiple ^131^I treatments at higher doses (100–200 mCi), causing an increase in the number of adverse reactions in the hematopoietic system, gastrointestinal system, and even marrow depression [3]. Due to the risk factors for ^131^I therapy-induced injury, increasing attention has been paid to efficacious radioprotective agents to combat radionuclide toxicity.

The intestinal mucosa is the fastest self-renewing tissue, which contributes to its high sensitivity to radiation, and the gut microbiota consists of trillions of microorganisms and is possibly involved in the response to radiation damage [4]. It is well-known that gut microbiota can exert its effects on radiation responses by producing bioactive compounds, especially short-chain fatty acids (SCFAs) [5, 6]. In addition, the gut microbiota can also regulate the biosynthesis and uptake of polyunsaturated fatty acids (PUFAs) [7]. During different types of PUFA for the treatment of intestinal regeneration after radiation, arachidonic acid (ARA) treatment could serve as a direct proliferation promoter of intestinal epithelial cells [8]. As one of the most abundant PUFAs in human tissues, ARA is typically esterified to membrane phospholipids, presents in all mammalian cells, and serves as a substrate for many enzymatic transformations that produce biologically active lipid mediators including prostaglandins, leukotrienes, epoxyeicosatrienoic acids, and endocannabinoids. Along with its metabolites, ARA is broadly involved in numerous physiological functions such as promoting cell proliferation, angiogenesis, and inflammation [9]. Paradoxically, some ARA-derived metabolites have been recognized to play important roles in the remission of inflammation [10]. These considerations prompted us to further investigate the crucial role between gut microecology and ARA in ionizing radiation (IR).

**Fig. 1:**
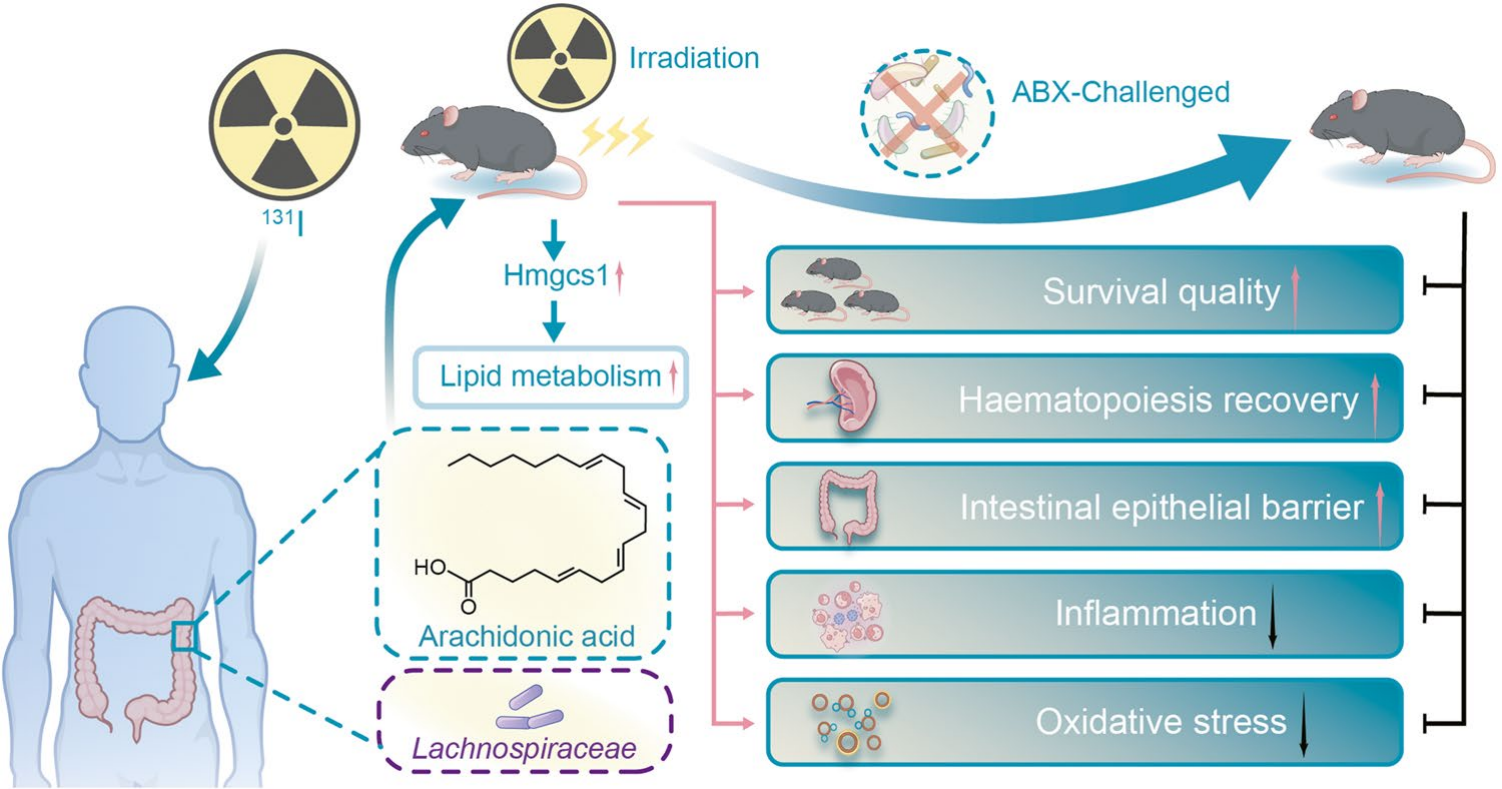
Microbiota-metabolite axis plays a pivotal role in radionuclide protection. *Lachnospiraceae* and arachidonic acid (ARA) metabolites significantly contribute to radioprotection after ^131^I therapy. ARA treatment in mice alleviated irradiation toxicity by regulating inflammation and oxidative stress, restoring hematopoiesis, and regenerating intestinal epithelial cells by activating hydroxy-3-methylglutaryl-coenzyme A synthase 1 (Hmgcs1) and enhancing lipid metabolism. ABX, antibiotic

In our study, we first explored alterations in the gut microbiota and metabolites after high-dose ^131^I therapy (100–150 mCi) using multi-omics analyses (microbiome analysis and untargeted and targeted metabolomics). Subsequently, we hypothesized that treatment with ARA could attenuate IR-induced injuries in mice by improving the quality of life, recovering hematopoiesis and gastrointestinal systems, regulating inflammation and oxidative stress, and saving the natural intestinal microbiome and metabolites (metagenomics and untargeted metabolomics). We further explored the underlying mechanisms of ARA treatment using proteomics to validate its radioprotective effects. Our research provides a better understanding of the function and possible radioprotective mechanism of the microbiota and metabolites in the context of radionuclide toxicity in preclinical settings (Fig. 1).

## Materials and methods

### Study subjects and design

This study recruited 102 DTC patients (33 males and 69 females, ages 23–55 years) undertaking ^131^I therapy (100–150 mCi, oral) from the Department of Nuclear Medicine, Shanghai Tenth People’s Hospital, between March and July 2021. DTC diagnosis was based on clinical guidelines within 6 months [11]. Any patient suffering from gastrointestinal disease, any other severe physical or mental disorder, or oral administration of probiotics or antibiotics were excluded. This study was conducted in accordance with the principles of the World Medical Association and the Declaration of Helsinki and was approved by the Ethics Committee of the Shanghai Tenth People’s Hospital (No. SHSY-IEC-5.0/22K13/P01). Written informed consent was obtained from all patients.

### Fecal sample collection

Fecal samples were collected in the morning after an overnight fast (> 8 h) before and after treatment (3 days later). Fecal samples were snap-frozen with liquid nitrogen, collected, and stored at − 80 °C (Haier, DW-86L626, China) [12].

### Microbiomic, metabolomic, metagenomic, and proteomic analyses

All details on the sequencing methodology are shown in Supplemental Methods.

### Mice

Six- to eight-week-old male C57BL/6 J mice were purchased from the Beijing Vital River Laboratory Animal Technology Co., Ltd. (Beijing, China). The mice were housed in a specific pathogen-free (SPF) animal facility. Mice were kept in a controlled environment (22 °C ± 2 °C, 60% ± 5% relative humidity, and 12-h light/dark cycle) and provided with a standard diet and water. All animal experiments were approved by the Animal Ethics Committee of Shanghai Tenth People’s Hospital (SHDSYY-2021–2801), which complied with the Guide for the Care and Use of Laboratory Animals and the National Institutes of Health guide for the Care and Use of Laboratory Animals.

### Radiation

A Rad Source RS 2000 X-Ray Irradiator (Rad Source Technologies, Suwanee, GA, USA) at a dose rate of 1.0 Gy per minute was used for animal experiments. Mice treated with total body irradiation were exposed to 6 Gy (for survival quality experiment) and 4 Gy (for the remaining experiments) X-rays.

### Experimental treatments

All mice were acclimatized for 1 week and randomly assigned to different groups with corresponding treatments: (1) Con group: healthy 6–8-week-old C57BL/6 mice; (2) 4 Gy/6 Gy group: mice were exposed to 4 Gy/6 Gy irradiation; (3) 4 Gy/6 Gy + ARA group: mice were treated with ARA (Sigma, #A3611) [8] 400 mg/kg through oral gavage, dissolved in sterile water mixed with an ultrasound shaker for 5 min in 0.2 ml volume per mouse from 2 days before to 6 days after 4 Gy/6 Gy irradiation (interventions on alternate days). (4) ABX + 4 Gy + ARA group: Mice were treated for 14 days with antibiotic (ABX; vancomycin 100 mg/kg, Aladdin, Shanghai, China; neomycin 200 mg/kg, Aladdin, Shanghai, China; penicillin 200 mg/kg, Solarbio, Beijing, China; methenamine 200 mg/kg, Solarbio, Beijing, China) dissolved in sterile water via the oral route in a 0.2 ml volume per mice until 2 days before irradiation (interventions on alternate days). The mice were then treated with ARA, as mentioned above. (5) UA + 4 Gy + ARA group: Mice were treated with ursolic acid (UA) (Aladdin, Shanghai, China) 40 mg/kg through oral gavage, dissolved in sterile water mixed with ultrasound shaker for 5 min in a 0.2 ml volume per mouse every day from 2 days before to 6 days after 4 Gy irradiation. Mice were treated with ARA as mentioned above.

### Histopathology

Spleens and small intestines were collected and fixed in 10% neutral-buffered formalin. Then, 4-µm sections of paraffin-embedded samples were stained with hematoxylin and eosin (H&E, Sigma-Aldrich). Paraffin-embedded sections were dewaxed in xylene and ethanol before staining with Alcian Blue/periodic acid-Schiff (AB-PAS) C. Subsequently, the sections were washed with distilled water, acidified with AB-PAS B, and stained with AB-PAS A. Finally, the sections were washed with distilled water. In this study, samples were scored semi-quantitatively by a board-certified pathologist who was blinded to the experimental conditions.

### Blood analysis

Blood was drawn from each mouse and immediately mixed with EDTA at 4 °C for routine examination. Blood samples were analyzed using Celltac alpha MEK-6400 series hematology analyzer (Mindray BC-2800Vet, Shenzhen, China).

### ELISA analysis

Mouse blood samples were collected as described above and centrifuged (3000 rpm, 20 min) to obtain plasma. Secretion of mouse malondialdehyde (MDA) and IL-6 was measured using a Mouse MDA ELISA Kit (YC4320M) and Mouse IL-6 ELISA Kit (shycbio, YC3298M) according to the manufacturer’s instructions.

### Reverse transcription (RT)–quantitative PCR (RT-qPCR)

Total RNA was isolated using the Total RNA Isolation Kit V2 (Vazyme Biotech Co. Ltd. RC112). RNA was treated with DNase and converted to cDNA using HiScript^®^ III All-in-one RT SuperMix Perfect for qPCR (Vazyme Biotech Co. Ltd. R333-01). RT-qPCR was performed using the Taq Pro Universal SYBR qPCR Master Mix (Vazyme Biotech Co., Ltd. Q712) using a LightCycler^®^ 96 system (Roche). Analysis was performed using the 2(^−ΔΔCt^) method. The targeted genes were normalized by comparing their expression with that of GAPDH. Primer information is shown in Supplemental Methods.

### Statistical analysis

To assess significance, the mean values of independent groups were compared using Student’s *t*-test, one-way ANOVA, and Wilcoxon rank-sum test as follows: *p < 0.05; **p < 0.01; ***p < 0.001; ****p < 0.0001. Kaplan–Meier analysis was performed for survival analysis, and the significance between survival curves was determined using a log-rank test. Statistical significance was set at *p* < 0.05.

## Results

### ^131^I therapy changes the structure of the gut microbiota in patients with differentiated thyroid cancer

**Fig. 2:**
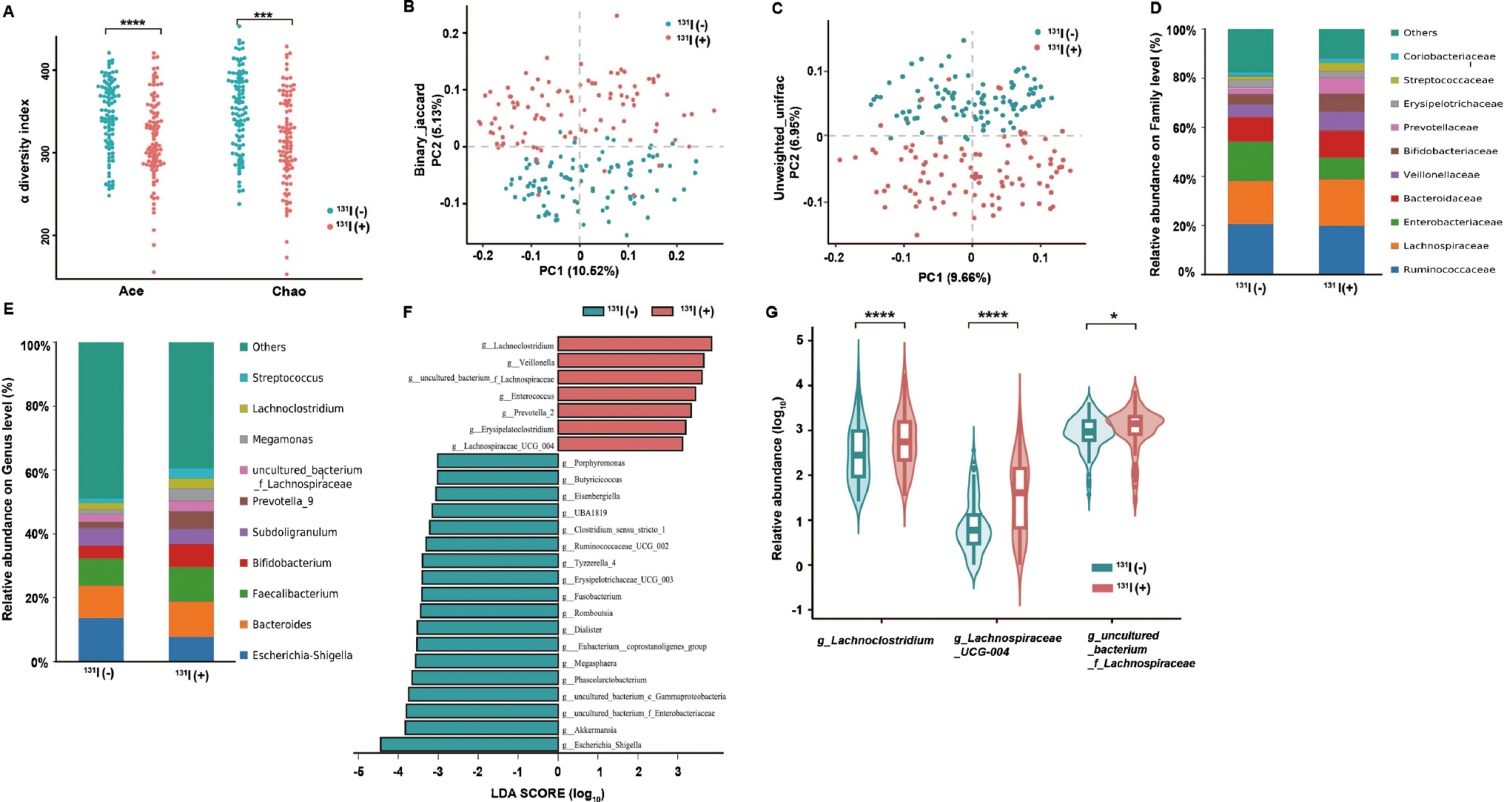
^131^I therapy changes the structure of gut microbiota in patients with DTC (*n* = 102). **A** α-diversity analysis in Ace and Chao1 indices between patients before ^131^I therapy [^131^I ( −)] and after ^131^I therapy [^131^I ( +)]. β-diversity analysis on principal coordinates (PC) analysis for binary_jaccard (**B**) and unweighted_unifrac (**C**) indices [^131^I ( +) vs ^131^I ( −)]. Abundance distribution on family (**D**) and genus (**E**) level [^131^I ( +) vs ^131^I ( −)]. **F** Linear discriminate analysis (LDA) effect size (LEfSe) revealing differential microbiota on genus level [^131^I ( +) vs ^131^I ( −)]. **G** Relative abundance of three genera in *Lachnospiraceae* identified by LefSe [^131^I ( +) vs.^131^I ( −)]

According to the flat rarefaction curves, sufficient sequencing data were gathered, and the amount of sequencing data was acceptable (Fig. S1A). In the diversity analysis, α-diversity assessed by the Ace and Chao1 indices of the gut microbiota was significantly decreased after ^131^I therapy (Fig. 2A). Both binary_jaccard and unweighted_uniFrac indices analyzed using principal coordinates analysis (PCoA) presented obvious changes (Fig. 2B, C; Fig. S1B, C), indicating that ^131^I therapy can greatly alter the composition of the gut microbiota. To further probe which bacterial taxa were distinct between the two groups, we compared the abundance distribution and found that the relative abundance of *Lachnospiraceae* was much higher at both family and genus levels (Fig. 2D, E). Additionally, linear discriminant analysis (LDA) effect size (LefSe) analysis at the genus level was performed to further corroborate the data on the representative taxa, and *Lachnospiraceae* was identified as the most predominant bacterial taxon after ^131^I treatment (Fig. 2F, G). Therefore, we speculated that *Lachnospiraceae* might play a crucial role in radiation protection after ^131^I treatment.

### ^131^I therapy alters the intestinal metabolite landscape of patients with DTC

**Fig. 3:**
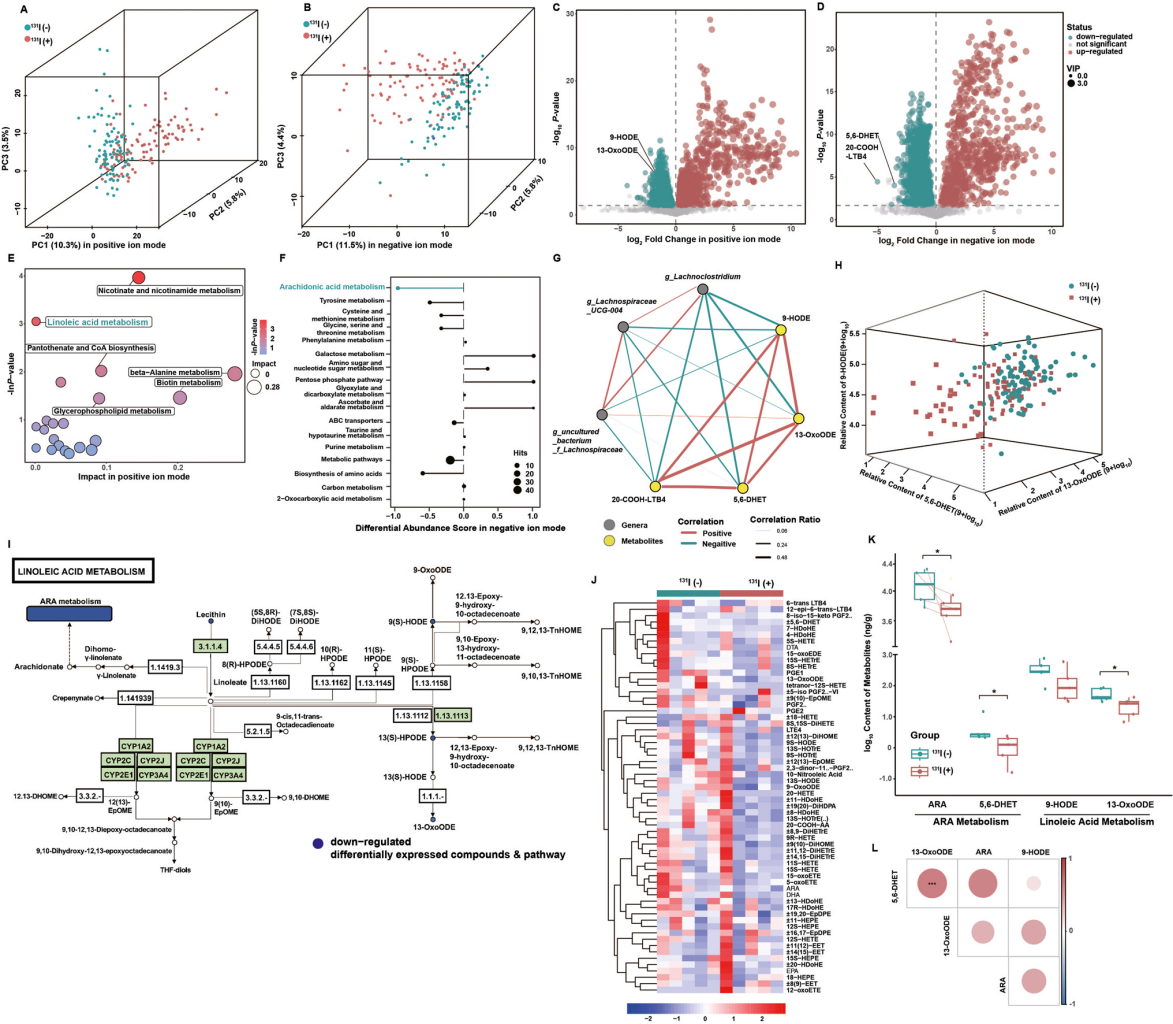
^131^I therapy alters the intestinal metabolite landscape of patients with DTC (*n* = 102). 3D principal components (PC) analysis for binary_jaccard index in positive (**A**) and negative (**B**) ion mode between patients before ^131^I therapy [^131^I ( −)] and after ^131^I therapy [^131^I ( +)]. Volcano plot of differential metabolites (fold change ≤ 0.5 or > 2) in positive (**C**) and negative (**D**) ion mode [^131^I ( +) vs ^131^I ( −)]. **E** Bubble plot of KEGG pathway analysis (most inhibited green marked) in positive ion mode [^131^I ( +) vs ^131^I ( −)]. **F** Differential abundance of KEGG pathways (most inhibited green marked) in negative ion mode [^131^I ( +) vs ^131^I ( −)]. **G** Spearman correlation network diagram among three core genera and four core lipid metabolites. **H** 3D scatter plot of three core lipid metabolites [^131^I ( +) vs ^131^I ( −)]. **I** KEGG metabolic pathways in LA metabolism [^131^I ( +) vs ^131^I ( −)]. **J** Heatmaps of targeted oxylipins contents [^131^I ( +) vs ^131^I ( −)]. **K** Box plots of alterations in ARA, ± 5,6 − DHET, 9-HODE, 13-OxoODE contents [^131^I ( +) vs.^131^I ( −)]. **L** Spearman correlation network diagram among ARA, ± 5,6 − DHET, 9-HODE, 13-OxoODE. *p < 0.05, ***p < 0.001

Next, we assessed the metabolome of the enteric flora to further explore the effects of ^131^I therapy on gut metabolites (Fig. 3). Orthogonal partial least-squares discrimination analysis (OPLS-DA) score plots (Fig. S2A-D) and principal components analysis (PCA) (Fig. 3A, B) revealed clear and separate clusters in the positive and negative ion modes between patients with DTC before and after ^131^I therapy. These results indicated that there were significant differences in the abundance of metabolites between the two groups, visualizing with volcano plots (Fig. 3C, D, more details in Supplemental Excel1). Compared with the before ^131^I therapy group, the differential metabolites with significantly decreased levels in positive ion mode were 9-HODE and 13-OxoODE in the after ^131^I therapy group (Fig. 3C). Pathway functional enrichment analysis showed that linoleic acid (LA) metabolism was robustly inhibited (Fig. 3E). In the negative ion mode, the metabolites with differentially decreased levels in the after ^131^I therapy group were 5,6-DHET and 20-COOH-LTB4 (Fig. 3D), which were found to be enriched in the ARA metabolism pathway by Kyoto Encyclopedia of Genes and Genomes (KEGG) pathway analysis (Fig. 3F).

Besides, heatmaps of the correlation between the levels of the top 45 differential metabolites (identified in the negative ion mode and plotted in a volcano plot) and the top 25 differential microbes by LefSe were analyzed (Fig. S2E). The abundance of *Lachnospiraceae* was negatively associated with the levels of LA metabolites (9-HODE and 13-OxoODE) and ARA metabolites (5,6-DHET and 20-COOH-LTB4) (Fig. 3G, Fig. S2F). The strong connection between LA and ARA metabolites was subsequently shown by a 3D scatter plot (Fig. 3H) and validated by the KEGG database (Fig. 3I in LA metabolism, Fig. S2G in ARA metabolism).

To further validate the results of non-targeted metabolomic analysis, especially those regarding ARA-/LA-related metabolites, we performed targeted metabolomic analysis of oxylipins in patients with DTC (*n* = 5) and identified 62 metabolites (Fig. 3J). Surprisingly, not only the content of LA metabolites (9-HODE and 13-OxoODE) and ARA metabolites (5,6-DHET) but also that of ARA was significantly lower in DTC patients after ^131^I therapy than in patients who were not treated (Fig. 3K). As expected, ARA metabolites (5,6-DHET) were strongly associated with LA metabolites (13-OxoODE) (Fig. 3L). Since ARA had a higher content in vivo and higher availability than ARA and LA metabolites (Fig. 3K) [13] and served as downstream of LA metabolism (Fig. 3I), we intended to explore the radioprotective effects of ARA instead of ARA metabolites or LA metabolites in our subsequent study.

### Oral gavage of ARA improves survival, decreases inflammatory response, and ameliorates hematopoietic and small intestine system injury in mice

**Fig. 4:**
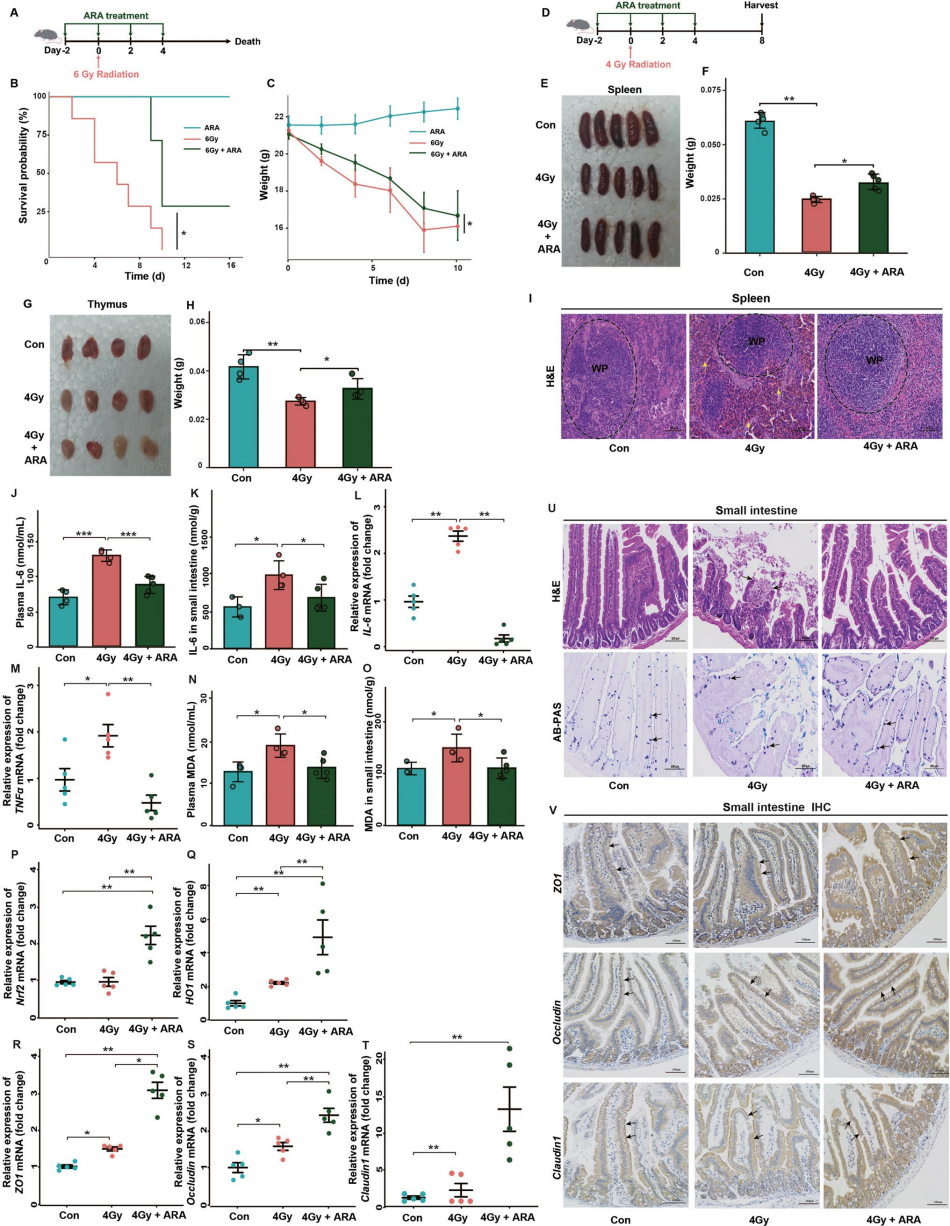
Oral gavage of ARA improves survival (6 Gy), decreases inflammatory response (4 Gy), and ameliorates hematopoietic and small intestine system injury (4 Gy) in mice. **A** Schematic of ARA treatment under 6 Gy irradiation. KM survival curves (**B**) and weight (**C**) for mice in 3 cohorts (*n* = 5 for ARA cohort; *n* = 7 for 6 Gy and 6 Gy + ARA cohorts, day 0 = the day of the irradiation). **D** Schematic of ARA treatment under 4 Gy irradiation. Photographs (**E**) and weight (**F**) of removed spleens from mice in 3 cohorts (*n* = 5 per cohort). Photographs (**G**) and weight (**H**) of removed thymuses from mice in 3 cohorts (*n* = 4 per cohort). **I** Spleens stained with H&E (× 200 magnification, congestion, yellow arrow). The content of IL-6 in plasma (**J**) and small intestine tissues (**K**) for mice in 3 cohorts assessed by ELISA (*n* = 5 per cohort). The IL-6 (**L**) and TNFα (**M**) levels in small intestine tissues for mice in 3 cohorts assessed by q-PCR (*n* = 5 per cohort). The content of MDA in plasma (**N**) and small intestine tissues (**O**) for mice in 3 cohorts assessed by ELISA (*n* = 5 per cohort). The Nrf2 (**P**), HO1 (**Q**), ZO1 (**R**), occludin (**S**), and claudin1 (**T**) levels in small intestine tissues for mice in 3 cohorts assessed by q-PCR (*n* = 5 per cohort). The small intestines stained with H&E (× 200 magnification, broken intestinal epithelium, black arrow), AB-PAS (× 200 magnification, goblet cells, black arrow) (**U**), and IHC (× 200 magnification, stained with antibodies, black arrow) (**V**). *p < 0.05, **p < 0.01; WP, white pulp

We then performed animal studies to verify whether ARA, identified as a potential radioprotective metabolite in our clinical studies, might have relevant effects in mice. The first goal of our study was to determine whether ARA supplementation could decrease the mortality rate associated with radiation (Fig. 4A) [14]. Body weight loss is an effect of radiation-related toxicity [15], and oral administration of ARA did not decrease the survival rate or body weight, suggesting that ARA caused no detectable harm to the mice (Fig. 4B, C). Following 6 Gy whole-body irradiation, the survival rate and body weight were much higher in the ARA treatment group than in the control (Con) vehicle group (Fig. 4B, C), validating that radiation-induced mortality and weight loss can be prevented by ARA replenishment.

Since the hematopoietic system is particularly susceptible to radiation, we subsequently explored the radioprotective function of ARA in the hematopoietic system (Fig. 4D) [14]. With exposure to 4 Gy irradiation, the size and weight of the thymus and spleen of mice were significantly reduced but could be partially restored by ARA treatment (Fig. 4E–H). H&E pathological analyses implied that irradiation contributed to damage to the splenic white pulp (WP), but no tissue damage of the splenic WP area was showed in the ARA group (Fig. 4I). Analysis of routine blood test confirmed that white blood cell (WBC) counts, platelet (PLT) counts, and percentage of lymphocytes (LY%) were obviously decreased, but the percentage of neutrophil granulocytes (NE%) was increased in the irradiation group. The opposite results were observed after ARA replenishment (Fig. S3A-D).

In addition to the hematopoietic system, moderately high doses of radiation affect systemic inflammation and the gastrointestinal system [14]. Subsequently, we evaluated the protective effects of ARA on radiation-induced inflammation and gastrointestinal injury (Fig. 4D). Following ARA administration, mice exposed to 4 Gy irradiation exhibited reduced levels of inflammatory cytokines in the plasma and small intestine tissues (Fig. 4J–M), suggesting that ARA ameliorated radiation-induced systemic and intestinal inflammation. We next investigated whether ARA mediated radioprotection in mice by preventing irradiation-induced oxidative stress [16]. After ARA treatment, high oxidative stress levels induced by radiation were recovered, with decreased level of *MDA* (oxidative stress marker) and increased level of *Nrf2* and *HO1* (antioxidant markers) (Fig. 4N–Q). We further measured the expression of *ZO1*, *occludin*, and *claudin1*, which are regulators of epithelial proliferation and survival after toxic stimuli [17–19]. It was shown that the level of *ZO1*, *occludin*, and *claudin1* increased after ARA treatment, indicating that ARA could promote intestinal regeneration and maintenance of the mucosal epithelial barrier (Fig. 4R–T), as verified by H&E (of intestinal epithelial barrier integrity), AB-PAS (for intestinal epithelial regenerative ability), and immunohistochemistry (IHC) (for intestinal epithelial tight junction proteins) staining in small intestine tissues (Fig. 4U, V).

### ARA retains gut bacteria and metabolite composition influenced by irradiation

**Fig. 5:**
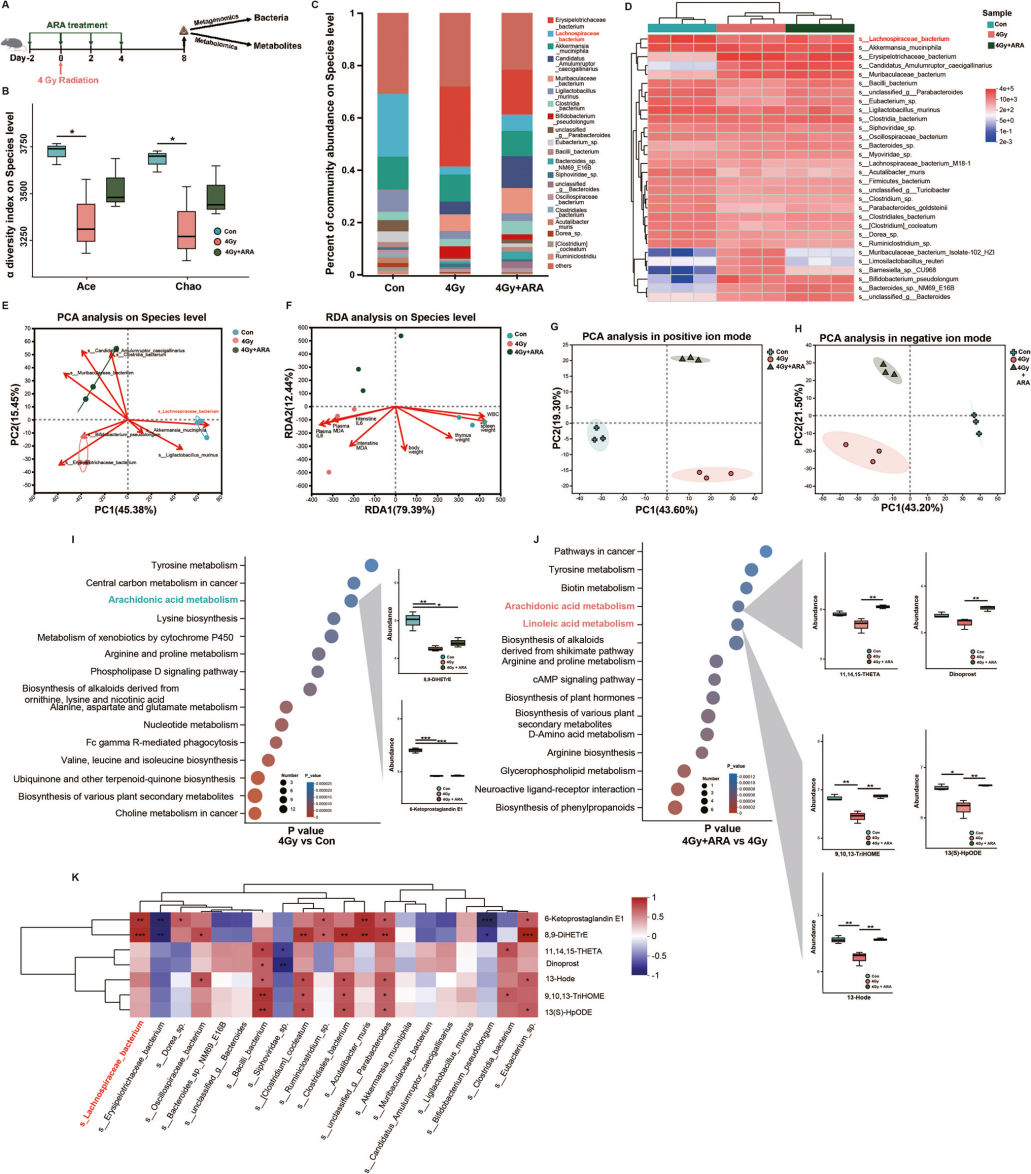
ARA retains gut bacteria and metabolite composition influenced by irradiation (4 Gy). **A** Schematic of fecal collection for bacteria and metabolite sequencing after ARA treatment under 4 Gy irradiation (*n* = 3 per cohort). **B** α-diversity analysis in Ace and Chao1 on species level in 3 cohorts. **C** Abundance distribution on species level (top 20) in 3 cohorts (s_Lachnospiraceae red marked). **D** Heatmap analysis on species level (top 30) in 3 cohorts (s_Lachnospiraceae red marked). **E** Principal components (PC) analysis (PCA) on species level in 3 cohorts (s_Lachnospiraceae red marked). **F** Redundancy analysis (RDA) and clinical factors on species level in 3 cohorts. **G** PCA analysis in positive ion mode in 3 cohorts. **H** PCA analysis in negative ion mode in 3 cohorts. KEGG Enrichment Analysis (**I**, 4 Gy vs control; **J**, 4 Gy + ARA vs 4 Gy; inhibited pathways green marked; activated pathways red marked). **K** Spearman correlation between seven metabolites enriched in ARA metabolism and LA metabolism and top 20 species (s_Lachnospiraceae red marked). *p < 0.05, **p < 0.01, ***p < 0.001

Based on the close association between intestinal microecology and radiation progression mentioned above, we assessed the therapeutic effects of ARA treatment on alterations in gut bacteria (metagenomics) and metabolites (non-targeted metabolomics) under 4 Gy irradiation in mice (Fig. 5A). The α-diversity analysis of the ACE and Chao1 indices showed that the abundance of gut microbiota was significantly decreased after irradiation, but this damage was partly attenuated by ARA replenishment (Fig. S4A at the genus level and Fig. 5B at the species level). We next compared the microbiota abundance distribution among the three cohorts and found that *Lachnospiraceae* was the most significant bacteria that showed low abundance in the irradiation group and recovered its abundance in the ARA-applying group (Fig. S4B, C on genus level; Fig. 5C, D on species level; statistical tests in Fig. S4D, E). For β-diversity, the sample distance among the three cohorts was analyzed and plotted using PCA, and *Lachnospiraceae* was defined as the major contributor to the subject distribution, indicating that ARA might change the intestinal flora composition after radiation by regulating the abundance of *Lachnospiraceae* (Fig. S4F at the genus level and Fig. 5E at the species level). We enrolled clinical factors in mice for redundancy analysis (RDA), and the results showed that both inflammation and hematopoietic function factors were essential for the sample distribution (Fig. S4G at the genus level, Fig. 5F on species level). In metabolomic analysis, PCA in both positive and negative ion modes showed that ARA treatment could robustly alter the metabolite profiles after irradiation (Fig. 5G, H). KEGG enrichment analysis showed that differential metabolites between irradiation and control groups were enriched in ARA metabolism (8,9-DiHETrE and 6-ketoprostaglandin E1 decreased in the irradiation group) pathway (Fig. 5I); differential metabolites between ARA therapy and irradiation groups were enriched in ARA metabolism (11,14,15-THETA and dinoprost increased in ARA therapy group) and LA metabolism (9,10,13-TriHOME, 13(S)-HpODE and 13-HODE increased in ARA therapy group) pathways (Fig. 5J), consistent with the metabolomic results analyzed in patients (Fig. 3E, F). The correlation heatmap also implied a highly positive connection between *Lachnospiraceae* and ARA metabolites (Fig. S4H at the genus level; Fig. 5K at the species level), showing that *Lachnospiraceae* as well as ARA and LA metabolism was the most predominant bacteria and metabolites affected by ARA treatment under 4 Gy irradiation.

### The gut microbiota impacts the radioprotective effects of ARA treatment in mice

**Fig. 6:**
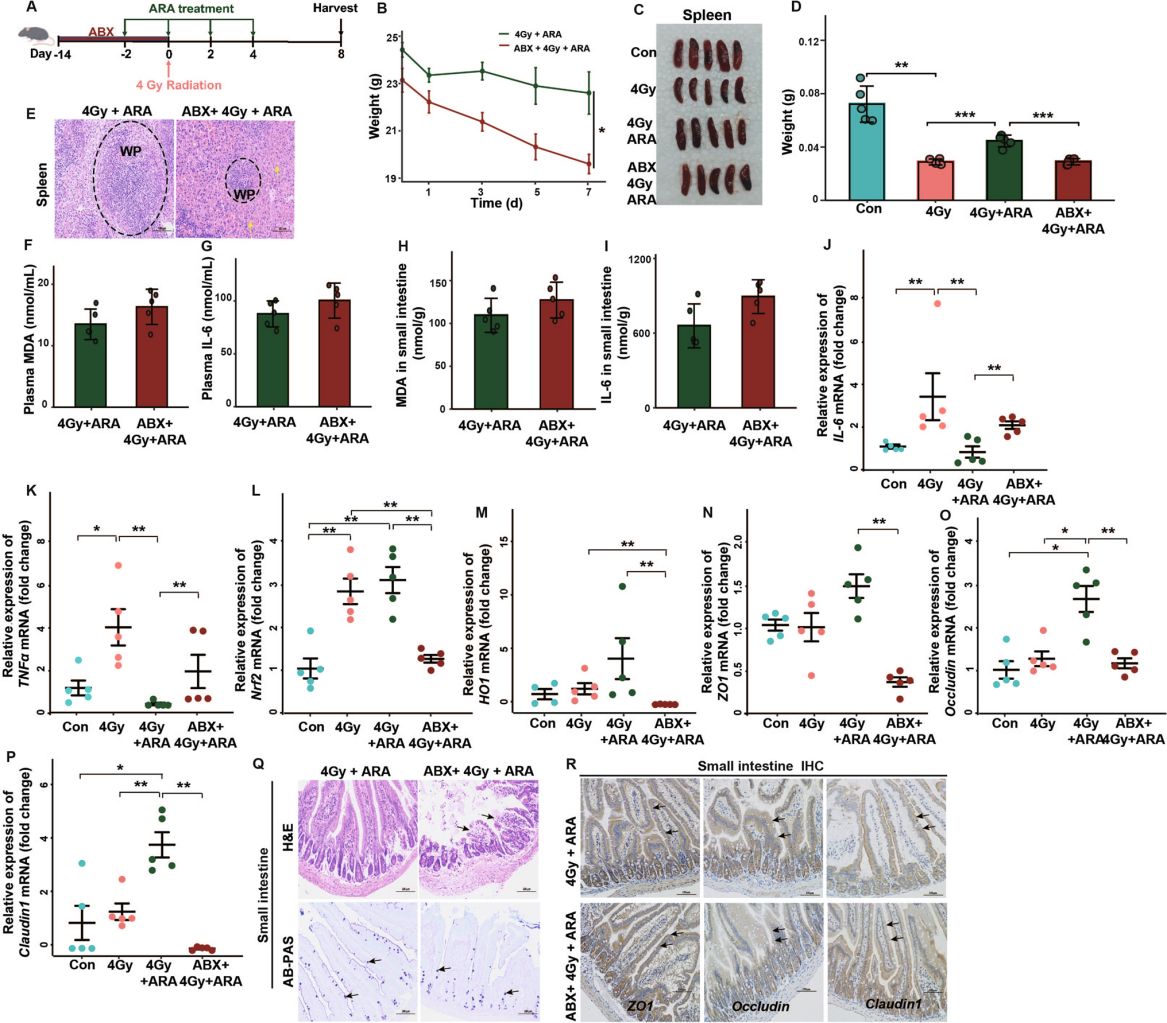
Gut microbiota impacts the radioprotective effects (4 Gy) of ARA treatment in mice. **A** Schematic of antibiotics (ABX) intervention before ARA treatment. **B** The weight for mice in 2 cohorts (*n* = 4 for 4 Gy + ARA cohort; *n* = 5 for ABX + 4 Gy + ARA cohort, day 0 = the day of the irradiation). Photographs (**C**) and weight (**D**) of removed spleens from mice in 4 cohorts (*n* = 5 per cohort). **E** Spleens stained with H&E (× 200 magnification, congestion, yellow arrow). The content of plasma MDA (**F**) and IL-6 (**G**); MDA (**H**) and IL-6 (**I**) in small intestine tissues for mice in 2 cohorts assessed by ELISA (*n* = 5 per cohort). The IL-6 (**J**), TNFα (**K**), Nrf2 (**L**), HO1 (**M**), ZO1 (**N**), occludin (**O**), and claudin1 (**P**) levels in small intestine tissues for mice in 4 cohorts assessed by q-PCR (*n* = 5 per cohort). Small intestine stained with H&E (× 200 magnification, broken intestinal epithelium, black arrow), AB-PAS (× 200 magnification, goblet cells, black arrow) (**Q**),and IHC (× 200 magnification, stained with antibodies, black arrow) (**R**). *p < 0.05, **p < 0.01, ***p < 0.001

Given the aforementioned radioprotective effects of ARA treatment, we established an ABX treatment model to investigate whether ARA-moderated radiation toxicity is dependent on the gut microbiota (Fig. 6A). In the quality of life analysis, ARA treatment no longer alleviated body weight loss induced by 4 Gy irradiation in ABX-challenged mice (Fig. 6B). Furthermore, we evaluated the radioprotective function of ARA in the hematopoietic system. The results showed that ARA treatment failed to improve the mass of the spleen or promote hematopoietic recovery without the participation of intestinal flora (Fig. 6C–E). As expected, the loss of gut microbes made it difficult to modulate oxidative stress and inflammation via ARA treatment after irradiation (Fig. 6F–M). Moreover, ABX treatment eliminated the radioprotective effects of ARA replenishment by enhancing the mucosal barrier and protecting the intestinal epithelium (Fig. 6N–R). These findings suggest that ARA protects against radiation damage, possibly through the gut microbiota.

### ARA reconstructs the small intestinal protein expression profile and protects against radiation via Hmgcs1 in mice

**Fig. 7:**
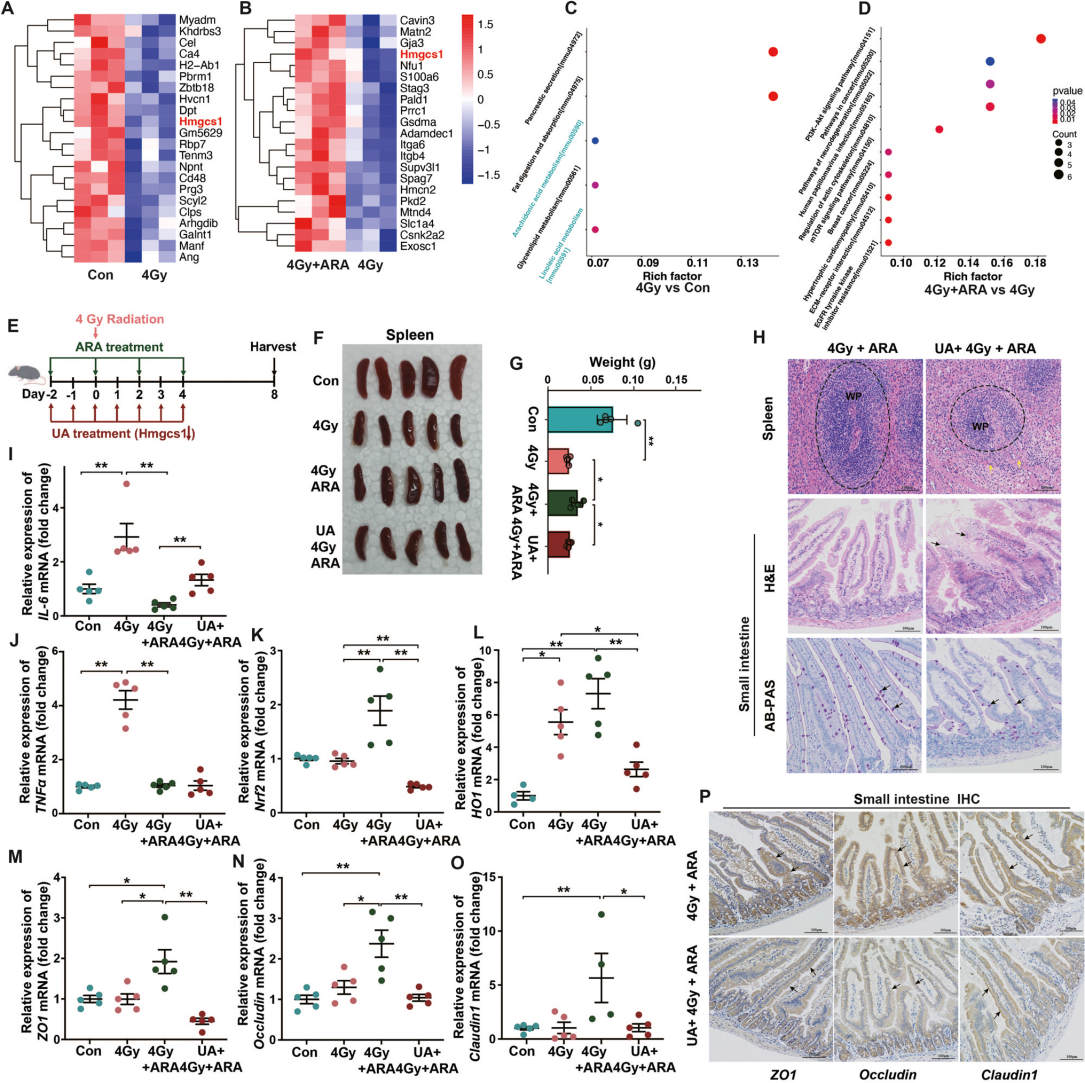
ARA reconstructs the small intestinal protein expression profile and protects against radiation (4 Gy) via Hmgcs1 in mice. Hierarchical cluster analysis for the differential proteins (fold change ≤ 0.6 or > 1.67) in the small intestine of mice in three cohorts (Hmgcs1 red marked) (**A**, control vs 4 Gy; **B**, 4 Gy + ARA vs 4 Gy; *n* = 3 per cohort). KEGG pathway enrichment analysis (most inhibited green marked) of differentially expressed proteins in the three cohorts (**C**, 4 Gy vs control; **D**, 4 Gy + ARA vs 4 Gy). **E** Schematic representation of ursolic acid (UA) intervention with ARA treatment. Photographs (**F**) and weight (**G**) of the spleens were removed from mice in four cohorts (*n* = 5 per cohort). **H** Spleens and small intestine stained with H&E (× 200 magnification, congestion, yellow arrow; broken intestinal epithelium, black arrow) and AB-PAS (× 200 magnification, goblet cells, black arrow). IL-6 (**I**), TNFα (**J**), Nrf2 (**K**), HO1 (**L**), ZO1 (**M**), occludin (**N**), and claudin1 (**O**) levels in the small intestine tissues of mice in the four cohorts assessed by q-PCR (*n* = 5 per cohort). **P** Small intestine stained with IHC (× 200 magnification, stained with antibodies, black arrow). *p < 0.05, **p < 0.01

To systematically assess the molecular mechanism of ARA treatment in radiation, we performed proteomic analysis of the small intestine tissues of mice from the Con and 4 Gy irradiation with or without ARA treatment groups (Con, 4 Gy, ARA + 4 Gy groups) to distinguish key regulatory proteins. Based on the screening criteria for differentially expressed proteins (DEPs) of *p* < 0.05 and fold change ≤ 0.6 or > 1.67, 64 significantly DEPs in the Con vs 4 Gy comparison and 86 significant DEPs in the ARA + 4 Gy vs 4 Gy comparison were identified (Fig. S5A, B; more details in Supplemental Excel2). According to the protein alterations identified by hierarchical cluster analysis, hydroxy-3-methylglutaryl-coenzyme A (HMG-CoA) synthase 1 (Hmgcs1) was the only protein downregulated in the 4 Gy group compared to the Con group, whereas it was upregulated in the ARA treatment group compared to the group treated with irradiation only (Fig. 7A, B), which was validated by qPCR (Fig. S5C), revealing that Hmgcs1 may serve as a target protein for ARA supplement. KEGG pathway enrichment analysis of DEGs revealed lower activation of ARA and LA metabolism in the irradiation-treated group than in the Con group (Fig. 7C), which was similar to the KEGG pathway results obtained through untargeted metabolomics (Figs. 3E, F and 5I). In the ARA + 4 Gy vs 4 Gy comparison, the PI3K–Akt signaling pathway and pathways in cancer were significantly stimulated by ARA treatment, suggesting that ARA might function as a radioprotectant in mice by promoting intestinal epithelial cell proliferation (Fig. 7D).

Based on the above analyses, we re-evaluated the radioprotective function of ARA treatment by inhibiting the expression of Hmgcs1 via UA (Fig. 7E, Fig. S5D) [20]. Interestingly, compared with only ARA treatment, the irradiated mice with Hmgcs1 silencing and ARA replenishment exhibited worse hematopoietic system and small intestine injuries, higher inflammation, and oxidative stress (Fig. 7F–P), suggesting that ARA provided radioprotection possibly dependent on the Hmgcs1 target.

## Discussion

According to a limited number of safe and effective agents available in DTC clinical practice against ^131^I radiotoxicity, our study focused on the characteristics of the gut microbiota and metabolites in DTC patients with ^131^I therapy and validated their protective effects against IR. Our diversity analysis showed that ^131^I therapy decreased the abundance of microbes and remodeled them. We conclude that oral ^131^I therapy prompts radionuclides to sufficiently contact the intestinal mucosa and easily change the flora composition. Notably, despite the reduced abundance of most microbes, the proportion of *Lachnospiraceae* showed a striking increase at the family and genus levels and served as the dominant bacteria after ^131^I treatment. Studies have shown that *Lachnospiraceae* is the major regulator of host defense against high-dose radiation by influencing the metabolism of SCFAs (propionate and butyrate) [21, 22]. Butyrate, which is mainly generated by *Lachnospiraceae*, has been suggested for use in patients with IR-induced diarrhea because it can moderate gut-associated inflammation [23]. Therefore, we speculated that *Lachnospiraceae* might play a major role in modulating disease susceptibility after ^131^I radiation challenge.

Metabolites were also significantly affected by ^131^I radiation. Contrary to our assumptions, ARA- and LA-derived metabolites, instead of SCFAs, showed extreme radiosensitivity to ^131^I interventions, which was further confirmed by oxylipin metabolomics and proteomics and was closely linked to *Lachnospiraceae*. Similarly, the abundance of *Lachnospiraceae *was significantly increased, which was accompanied by high enrichment of ARA metabolism in an aging model [24]. Our animal study also verified that ARA supplementation increased the abundance of *Lachnospiraceae*. Conversely, the abundance of *Lachnospiraceae* was lower in the microbiota of patients with hematochezia with chronic radiation proctitis and activated ARA metabolism [25]. Based on the studies presented above, we hypothesized that ARA exerted its radioprotective effects, partly based on the enteric microbiota. To address this assumption, the microbes in the mice were removed using ABX. Indeed, ABX-challenged mice did not respond to ARA treatment and exhibited toxicity similar to that observed in mice treated with irradiation only, indicating that ARA was dependent on gut microbiome function. Further studies are required to explore whether and how *Lachnospiraceae* are involved in ARA metabolic processes.

Given that ARA displays multiple functions, our results examined the radioprotective effects of ARA on immunity and oxidative stress. Some ARA-derived metabolites serve as the potent anti-inflammatory mediator involved in resolving inflammation, such as lipoxin A4 and eicosanoids [26, 27]. In a systematic review of studies conducted in adults, increasing ARA intake to 1000–1500 mg/day did not adversely affect platelet aggregation, blood clotting, oxidative stress markers, or immune function [28]. These studies further support our findings regarding the alleviation of radiation-induced inflammation and oxidative stress in mice.

Some studies have also implied that ARA can activate the proliferation of intestinal stem cells [8]. ARA strongly promoted intestinal epithelial cell proliferation by increasing Ascl2 expression and activating the WNT signaling pathway, but inhibited intestinal epithelial cell differentiation after irradiation [8]. Moreover, ARA concentrations in individuals with colon cancer tissue were increased, suggesting that ARA has a proliferation-promoting effect in the intestine [29], which is similar to our results obtained through KEGG pathway enrichment analysis of DEGs associated with ARA treatment. Accordingly, further in vivo experiments are required to validate these mechanisms in follow-up studies.

Finally, we discovered and validated Hmgcs1 as a potential major downstream target of ARA. Hmgcs1 plays a key role in cholesterol metabolism by converting acetoacetyl-CoA to HMG-CoA [30]. Our research showed the downregulation of Hmgcs1 in post-radiation intestinal tissues, suggesting perturbation of cholesterol biosynthesis by radiation. Based on these results and the comparison of pathways between the Con and radiation groups involved in the inhibition of ARA metabolism and LA metabolism through metabolomics and proteomics, we speculated that radiation might result in lipid metabolism disorders. Furthermore, stimulated Hmgcs1 exerts a radioprotective effect on cervical cancer cells. In contrast, the loss of Hmgcs1 by shRNAs increases the sensitivity to radiation [31]. Therefore, whether ARA participates in the regulation of lipid metabolism after radiation and the regulatory mechanisms between ARA and Hmgcs1 require further investigation.

Our study has several limitations regarding the experimental design and exploration of the mechanism. First, we did not observe the radioprotective function of *Lachnospiraceae* or fecal microbiota transplantation in mice subjected to irradiation. Second, ARA treatment experiments and microbiotic and metabolic sequencing were not repeated in female mice. In addition, future studies should investigate the relationship among ARA metabolites, lipid metabolism, and radiation protection.

In conclusion, the characteristics of the gut microbiota and metabolites in patients with DTC are remarkably transformed after ^131^I therapy, and ARA treatment can prevent IR-induced injury in mice. *Lachnospiraceae* and ARA metabolites significantly contribute to radioprotection. ARA supplementation in mice ameliorates irradiation toxicity by controlling inflammation and oxidative stress, recovering hematopoiesis, promoting intestinal epithelial regeneration, and preserving intestinal bacteria and metabolite composition. Additionally, ARA is involved in lipid metabolism by stimulating enteric Hmgcs1 to perform its radioprotective functions. ARA may be recognized as a therapeutic agent for treating the adverse side effects of radionuclides.

### Supplementary Information

Below is the link to the electronic supplementary material.

Supplementary file1 (JPG 248 KB)

Supplementary file2 (JPG 1776 KB)

Supplementary file3 (JPG 351 KB)

Supplementary file4 (JPG 2809 KB)

Supplementary file5 (JPG 480 KB)

Supplementary file6 (XLSX 503 KB)

Supplementary file7 (XLSX 49 KB)

Supplementary file8 (DOCX 17 KB)

Supplementary file9 (DOCX 29 KB)

## Data Availability

The original data presented in this study are available from the corresponding authors upon reasonable request.
