# Supplementary figures and images for: Disrupted gut microecology after high-dose 131I therapy and radioprotective effects of arachidonic acid supplementation
Source: Eur J Nucl Med Mol Imaging. 2024 Apr 2;51(8):2395–408. doi: 10.1007/s00259-024-06688-9 (PMC11178657; doi:10.1007/s00259-024-06688-9)

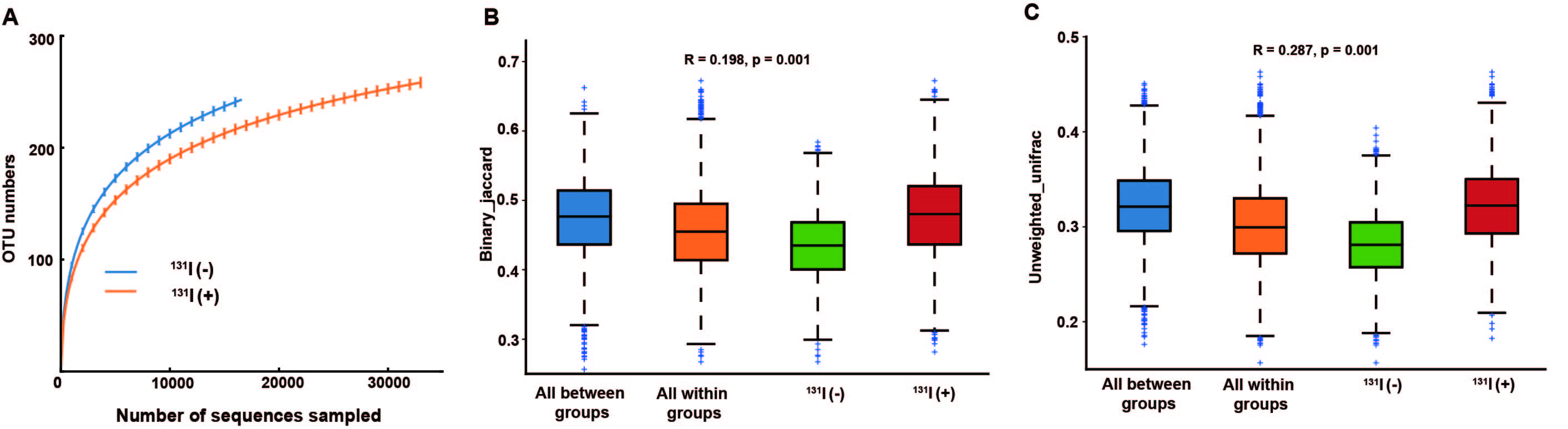

Supplement: Supplementary file 1 — Supplementary file1 (JPG 248 KB) [file 259_2024_6688_MOESM1_ESM.jpg]

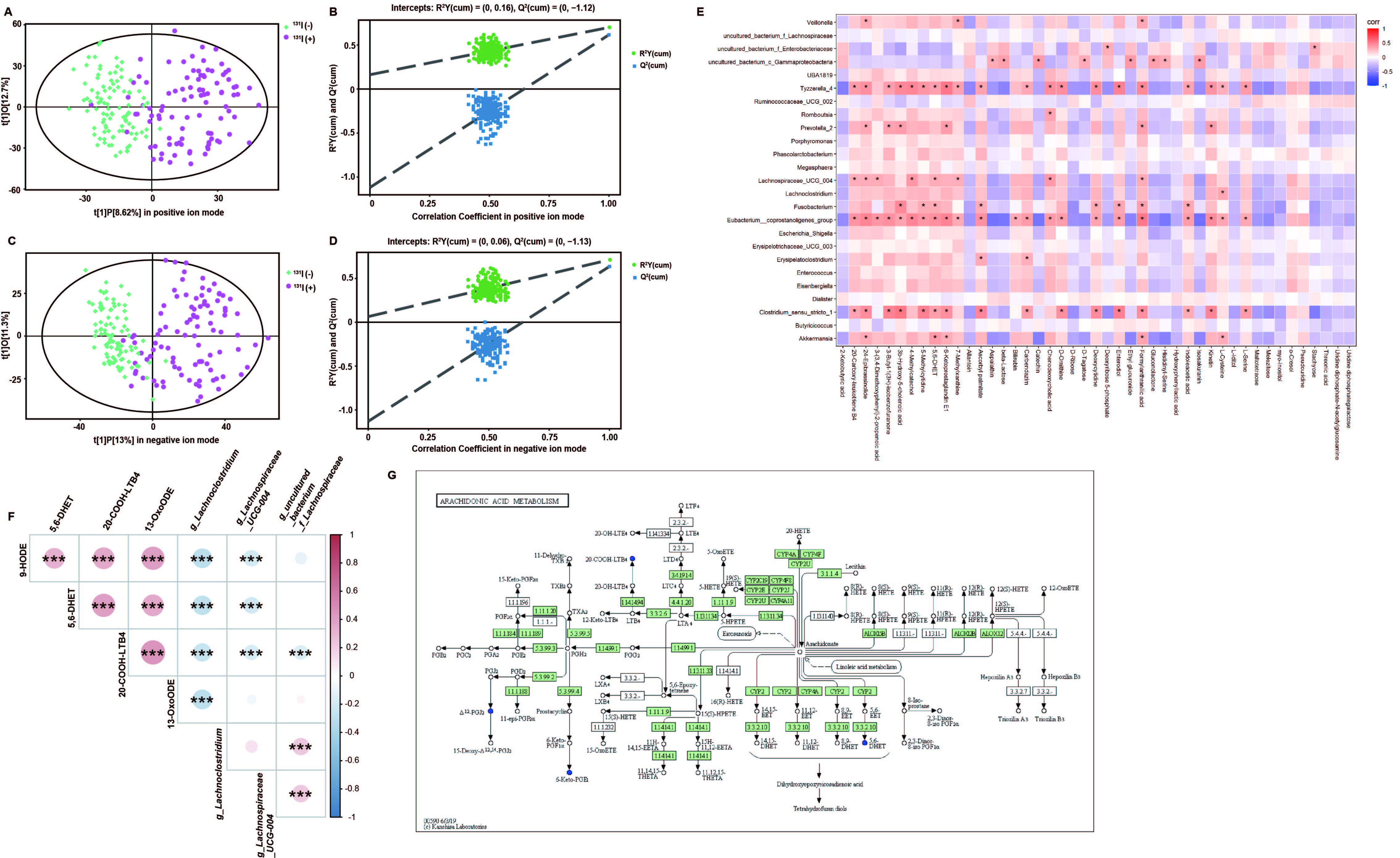

Supplement: Supplementary file 2 — Supplementary file2 (JPG 1776 KB) [file 259_2024_6688_MOESM2_ESM.jpg]

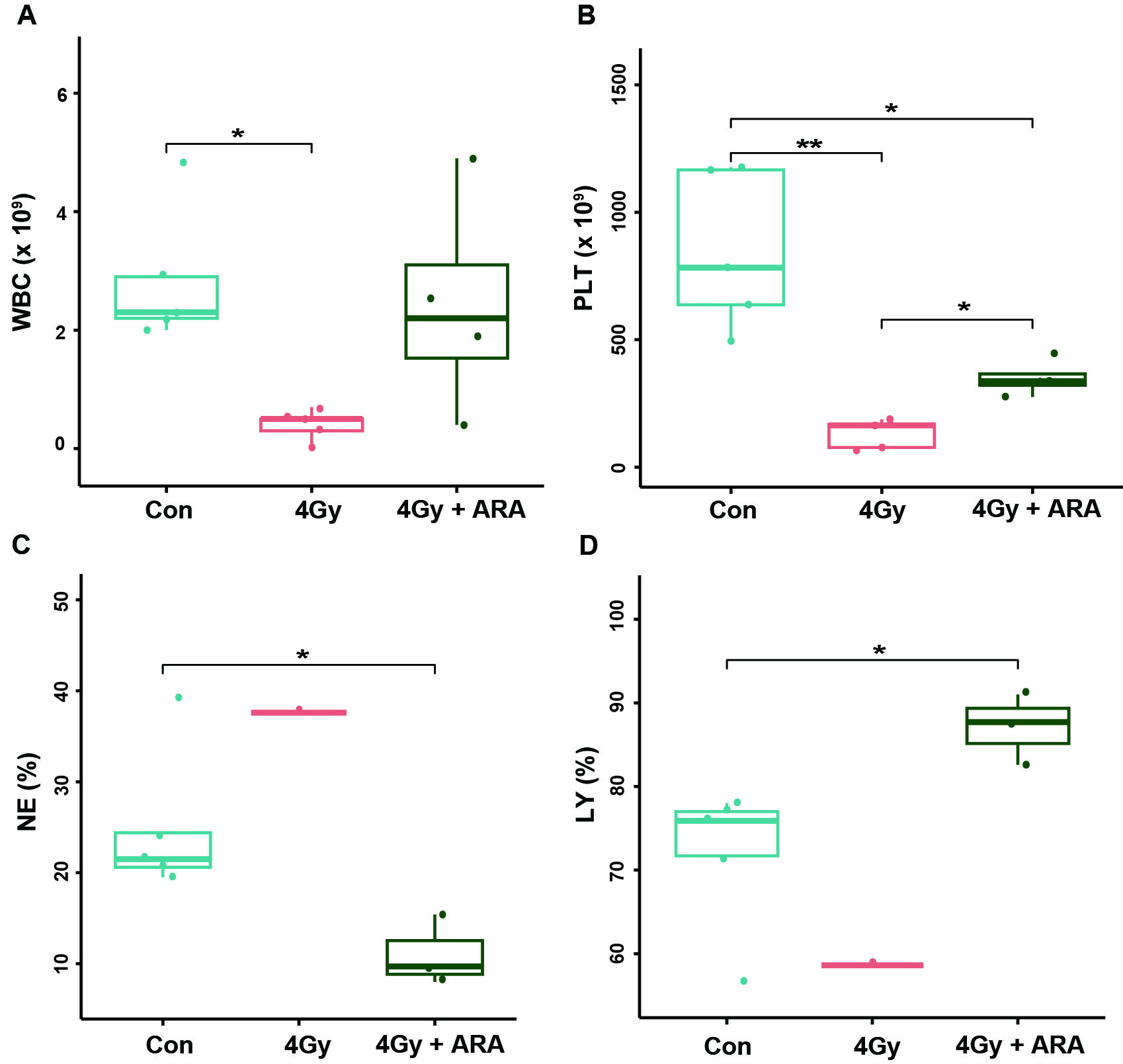

Supplement: Supplementary file 3 — Supplementary file3 (JPG 351 KB) [file 259_2024_6688_MOESM3_ESM.jpg]

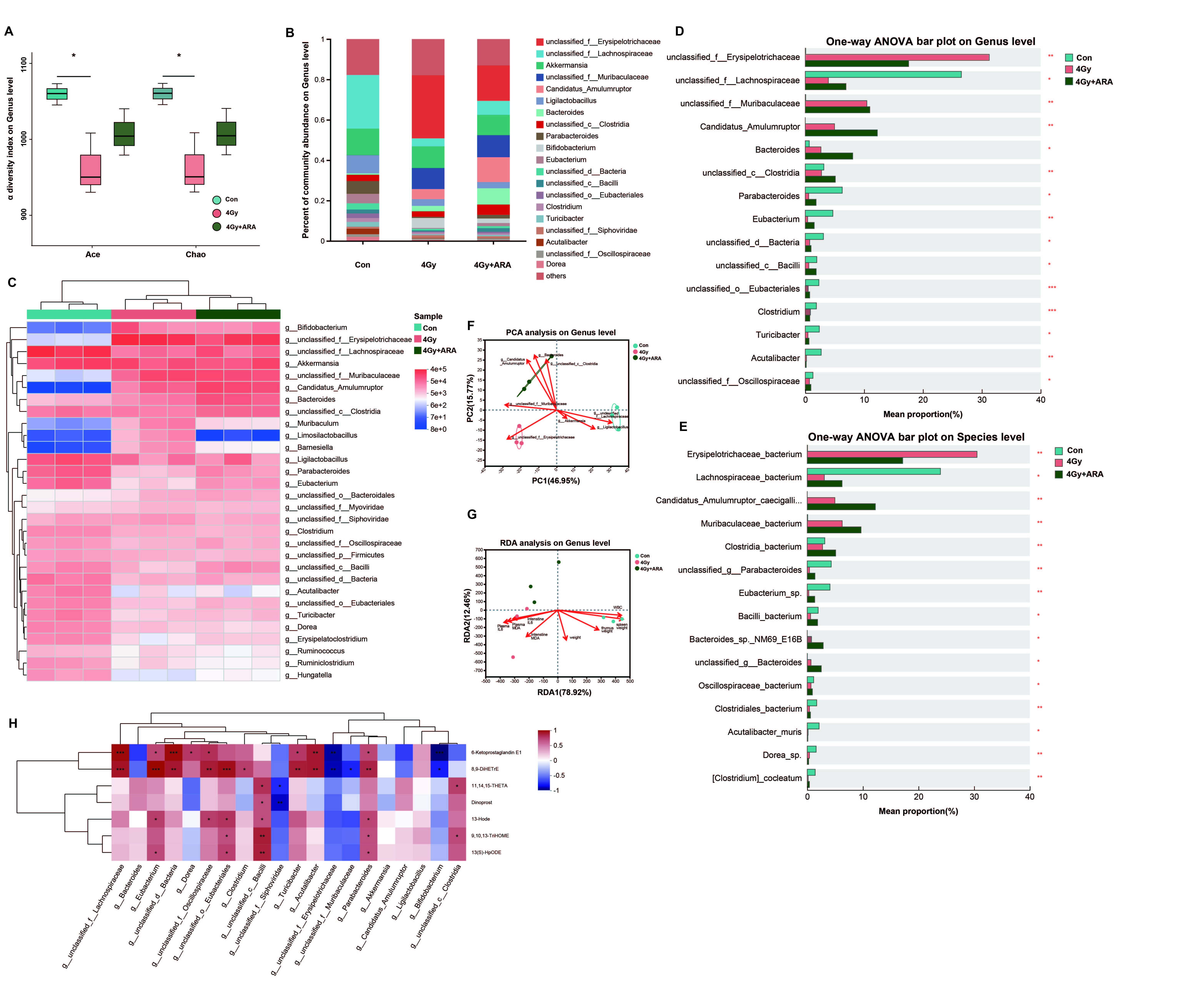

Supplement: Supplementary file 4 — Supplementary file4 (JPG 2809 KB) [file 259_2024_6688_MOESM4_ESM.jpg]

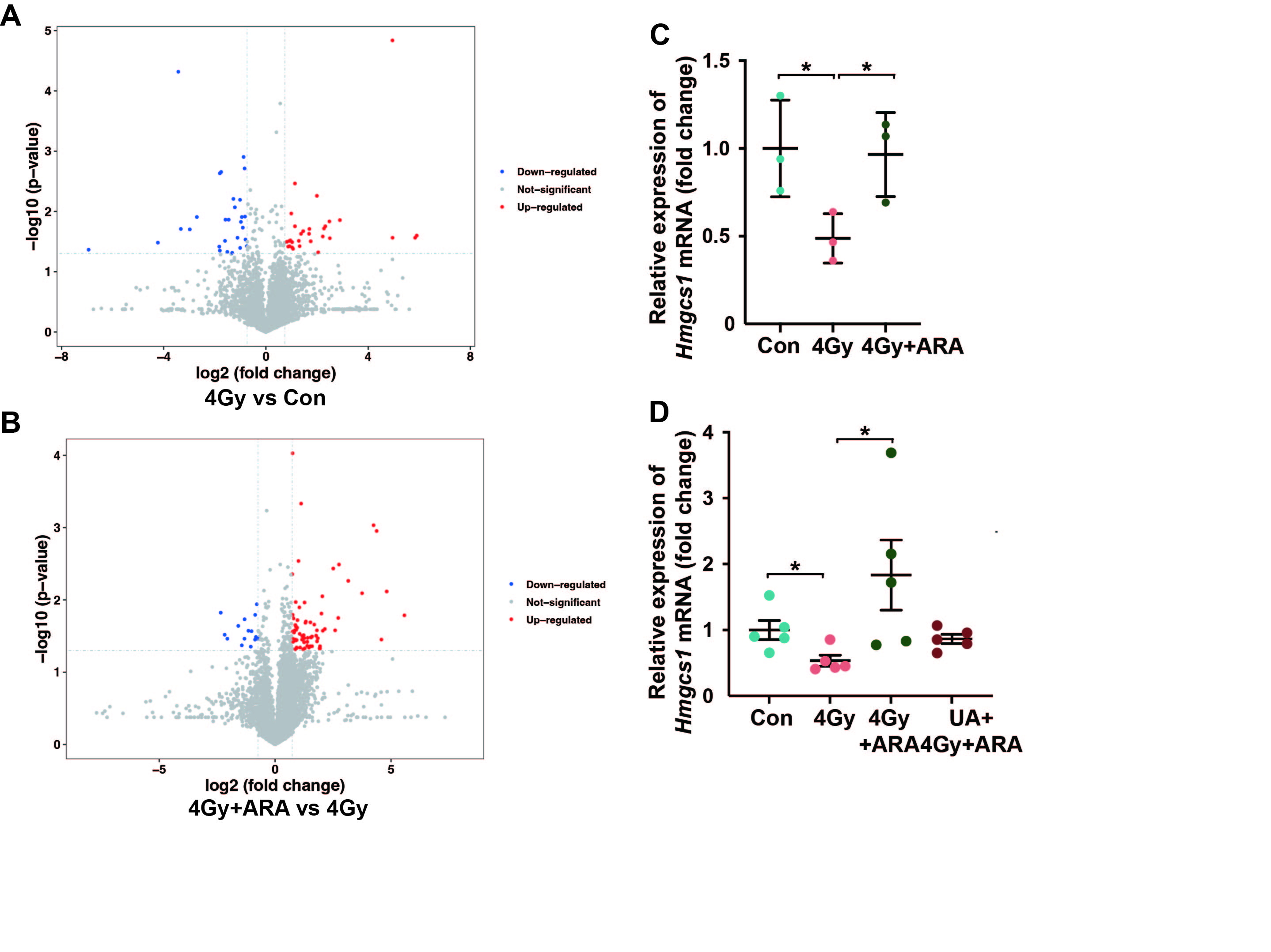

Supplement: Supplementary file 5 — Supplementary file5 (JPG 480 KB) [file 259_2024_6688_MOESM5_ESM.jpg]
