# Supplementary material for: Disrupted gut microecology after high-dose 131I therapy and radioprotective effects of arachidonic acid supplementation
Source: Eur J Nucl Med Mol Imaging. 2024 Apr 2;51(8):2395–408. doi: 10.1007/s00259-024-06688-9 (PMC11178657; doi:10.1007/s00259-024-06688-9)
Supplement: Supplementary file 8 — Supplementary file8 (DOCX 17 KB) [file 259_2024_6688_MOESM8_ESM.docx]

**Supplemental Figure Legends**

**Fig. S1 ^131^I therapy changes the structure of gut microbiota in patients with DTC (n=102).**

(**A**) α diversity analysis in rarefaction curves between patients before ^131^I therapy [^131^I (-)] and after ^131^I therapy [^131^I (+)]. PERMANOVA based on binary_jaccard distance (**B**) and unweighted_unifrac distance (**C**).

**Fig. S2** **^131^I therapy alters the intestinal metabolite landscape of patients with DTC (n=102).**

OPLS-DA score plot in positive(**A**) and negative (**C**) ion mode between patients before ^131^I therapy [^131^I (-)] and after ^131^I therapy [^131^I (+)]. Permutation test of OPLS-DA model in positive(**B**) and negative (**D**) ion mode. (**E**) Heatmaps of correlation between top 25 differential microbiota by LEfSe and top 45 differential metabolites by volcano plot in negative ion mode. (**F**) Statistical tests of correlation analysis among 3 core genera and 4 core lipid metabolites. (**G**) KEGG metabolic pathways in arachidonic acid metabolism [^131^I (+) vs ^131^I (-)] (down regulated differentially expressed compounds blue marked). * *p* < 0.05, *** *p* < 0.001.

**Fig. S3 Oral gavage of ARA improves survival, decreases inflammatory response and ameliorates haematopoietic and small intestine system injury in mice**

Peripheral blood examination of white blood cell (WBC) (**A**) and platelet (PLT) (**B**) counts and percentage of neutrophils (NE%) (**C**) and lymphocytes (LY%) (**D**) in 3 cohorts (n=5 per cohort, NE% and LY% expression not detectable in some peripheral blood samples). * *p* < 0.05, ** *p* < 0.01.

**Fig. S4 ARA retains gut bacteria and metabolites composition influenced by irradiation.** (**A**) α diversity analysis in Ace and Chao1 on Genus level in 3 cohorts. (**B**) Abundance distribution on Genus level (top20) in 3 cohorts. One-way ANOVA bar plot on Genus (**C**) and Species (**D**) levels. (**E**) Heatmap analysis on Genus level (top30) in 3 cohorts. (**F**) PCA analysis on Species level in 3 cohorts (*s_Lachnospiraceae* red marked). (**G**) RDA analysis and clinical factors on Species level in 3 cohorts. (**H**) Spearman correlation between 7 metabolites enriched in ARA metabolism and linoleic acid metabolism and top 20 species. * *p* < 0.05, ** *p* < 0.01, *** *p* < 0.001.

**Fig. S5 ARA reconstructs the small intestinal protein expression profile and protects against radiation via Hmgcs1 in mice.**

(**A**) Volcano plot of DEPs (fold change ≤ 0.6 or > 1.67) in the Con vs. 4 Gy comparison. (**B**) Volcano plot of DEPs (fold change ≤ 0.6 or > 1.67) in the ARA + 4 Gy vs. 4 Gy comparison. (**C**) The Hmgcs1 levels in small intestine tissues for mice in 3 cohorts assessed by q-PCR (n=3 per cohort). (**D**) The Hmgcs1 levels in small intestine tissues for mice in 4 cohorts assessed by q-PCR (n=5 per cohort). * *p* < 0.05.
