# Supplementary material for: Disrupted gut microecology after high-dose 131I therapy and radioprotective effects of arachidonic acid supplementation
Source: Eur J Nucl Med Mol Imaging. 2024 Apr 2;51(8):2395–408. doi: 10.1007/s00259-024-06688-9 (PMC11178657; doi:10.1007/s00259-024-06688-9)
Supplement: Supplementary file 9 — Supplementary file9 (DOCX 29 KB) [file 259_2024_6688_MOESM9_ESM.docx]

**Supplemental Methods**

***Microbiomic analysis***

Bacterial DNA was extracted from fecal samples using a QIAamp Fast DNA stool Mini Kit (Qiagen, Cat# 51604) and amplified with barcoded bacterial primers targeting the 16S rRNA variable region 3–4 (V3–V4): forward primer 338F:5′-ACTCCTACGGGAGGCAGCA-3′ and reverse primer 806R:5′-GGACTACHVGGGTWTCTAAT-3′ [1]. Illumina NovaSeq6000 was used for the construction of sequencing libraries and paired-end sequencing of samples. Paired-end reads were merged using FLASH v1.2.7, and tags with more than six mismatches were removed [2]. Using Trimmomatic, tags with a quality score of 20 within a sliding window of 50bps were determined, and those shorter than 350bps were removed [3]. A total of 172 chimeras were removed from the denoised sequences and they were clustered into operational taxonomic units (OTUs) with a 97% similarity using the USEARCH (version 10.2) program. All OTUs were assigned a taxonomy by searching the Silva databases (Release128) using QIIME.

***Untargeted and targeted metabolomic analysis***

Fifty milligrams of fecal sample were weighed into an EP tube, and 1000 μL of extract solution (methanol: acetonitrile: water = 2:2:1, with isotopically labeled internal standard mixture) was added. The samples were then homogenized at 35 Hz for 4 min and sonicated for 5 min in an ice-water bath. Homogenization and sonication cycles were repeated for 3 times. The samples were then incubated for 1 h at -40 °C and centrifuged at 12000 rpm for 15 min at 4 °C. The resulting supernatant was transferred into a fresh glass vial for further analysis. A quality control (QC) sample was prepared by mixing an equal aliquot of the supernatant from all samples.

For targeted metabolism of oxylipins, the sample was further purified using SPE. The SPE cartridges were equilibrated with 1 mL methanol and 1 mL water. After loading the sample (supernatant obtained following the procedure described above), the cartridge was washed with 1 mL of 5% MeOH/H2O (v/v). The ﬂow-through fraction was discarded. Finally, the samples were eluted with 1 mL of MeOH, and then the eluent was evaporated to dryness under a gentle stream of nitrogen, and were reconstituted in 100 μL of 30% ACN/H2O (v/v). The reconstituted solution was vortexed for 30 s, homogenized at 60 Hz for 4 min, and sonicated for 5 min in an ice water bath. After centrifugation (1 min, 12000 rpm, 4 °C), the reconstituted solution was transferred to an EP tube with a filter membrane. After centrifugation (15 min, 12000 rpm, 4 °C), the clear supernatant was subjected to UHPLC-MRM-MS analysis.

LC-MS/MS (for untargeted metabolism) and UHPLC-MRM-MS (for targeted metabolism) analyses of metabolites were performed by Biotree Biomedical Technology Co., LTD (Shanghai, China).

LC-MS/MS analyses were performed using an UHPLC system (Vanquish, Thermo Fisher Scientific) with a UPLC BEH Amide column (2.1 mm × 100 mm, 1.7 μm) coupled to Q Exactive HFX mass spectrometer (Orbitrap MS, Thermo). The mobile phase consisted of 25 mmol/L ammonium acetate and 25 ammonia hydroxides in water (pH = 9.75) (A) and acetonitrile (B). The auto-sampler temperature was 4 ℃, and the injection volume was 3 μL. The QE HFX mass spectrometer was used for its ability to acquire MS/MS spectra on information-dependent acquisition (IDA) mode in the control of the acquisition software (Xcalibur, Thermo). In this mode, the acquisition software continuously evaluates the full scan MS spectrum. The ESI source conditions were set as following: sheath gas flow rate as 30 Arb, Aux gas flow rate as 25 Arb, capillary temperature 350 ℃, full MS resolution as 60000, MS/MS resolution as 7500, collision energy as 10/30/60 in NCE mode, spray Voltage as 3.6 kV (positive) or -3.2 kV (negative), respectively.

The raw data were converted to the mzXML format using ProteoWizard and processed with an in-house program, which was developed using R and based on XCMS, for peak detection, extraction, alignment, and integration. Then an in-house MS2 database (BiotreeDB) was applied in metabolite annotation. The cutoff for annotation was set at 0.3.

Stock solutions were individually prepared by dissolving or diluting each standard substance to give a final concentration of 1 μg/mL. An aliquot of each of the stock solutions was transferred to an Eppendorf tube form a mixed working standard solution. A series of calibration standard solutions were then prepared by stepwise dilution of this mixed standard solution (containing isotopically-labelled internal standard mixture in identical concentrations with the samples).

The UHPLC separation was carried out using an EXIONLC System (Sciex), equipped with a Waters ACQUITY UPLC BEH C18 column (150 × 2.1 mm, 1.7 μm, Waters). The mobile phase A was 0.01% formic acid in water, and the mobile phase B was 0.01% formic acid in acetonitrile. The column temperature was set at 50 ℃. The auto-sampler temperature was set at 4 ℃ and the injection volume was 10 μL.

A SCIEX 6500 QTRAP+ triple quadrupole mass spectrometer (Sciex), equipped with an IonDrive Turbo V electrospray ionization (ESI) interface, was applied for assay development. Typical ion source parameters were: Curtain Gas = 40 psi, IonSpray Voltage = -4500 V, temperature = 500 ℃, Ion Source Gas 1 = 30 psi, Ion Source Gas 2 = 30 psi.

The MRM parameters for each of the targeted analytes were optimized using flow injection analysis, by injecting the standard solutions of the individual analytes, into the API source of the mass spectrometer. Several most sensitive transitions were used in the MRM scan mode to optimize the collision energy for each Q1/Q3 pair (Table S2). Among the optimized MRM transitions per analyte, the Q1/Q3 pairs that showed the highest sensitivity and selectivity were selected as ‘quantifier’ for quantitative monitoring. The additional transitions acted as ‘qualifier’ for the purpose of verifying the identity of the target analytes.

SCIEX Analyst Work Station Software (Version 1.6.3) and Multiquant 3.03 software were employed for MRM data acquisition and processing.

***Metagenomic analysis***

The data were analyzed on the free online platform of Majorbio Cloud Platform (www.majorbio.com). Briefly, the raw sequencing reads were trimmed of adaptors, and low-quality reads (length<50 bp or with a quality value <20 or having N bases) were removed by fastp (https://github.com/OpenGene/fastp, version 0.20.0).Reads were aligned to the human genome by BWA (http://bio-bwa.sourceforge.net，version 0.7.17) and any hit associated with the reads and their mated reads were removed.

The quality-filtered data were assembled using MEGAHIT (<https://github.com/voutcn/megahit>, version 1.1.2). Contigs with a length ≥ 300 bp were selected as the final assembling result. Open reading frames (ORFs) from each assembled contigs were predicted using Prodigal (https://github.com/hyattpd/Prodigal，version2.6.3) and a length ≥ 100 bp ORFs were retrieved.

A non-redundant gene catalog was constructed using CD-HIT (http://weizhongli-lab.org/cd-hit/, version 4.7) with 90% sequence identity and 90% coverage. Gene abundance for a certain sample was estimated by SOAPaligner (https://github.com/ShujiaHuang/SOAPaligner, version soap2.21release) with 95% identity.

The best-hit taxonomy of non-redundant genes was obtained by aligning them against the NCBI NR database by DIAMOND (http://ab.inf.uni-tuebingen.de/software/diamond/, version 2.0.11) with an e-value cutoff of 1e-5. Similarly, the functional annotation (GO, KEGG, eggNOG, CAZy, CARD, PHI) of non-redundant genes was obtained. Based on the taxonomic and functional annotation and the abundance profile of non-redundant genes , the differential analysis was carried out at each taxonomic, functional, or gene-wise level by Kruskal–Wallis test.

***Proteomic analysis***

For each sample, 2 𝜇𝑔 of total peptides were separated and analyzed with a nano­UPLC (EASY-nLC1200) coupled to a Q Exactive HFX Orbitrap instrument (Thermo Fisher Scientific) with a nano­electrospray ion source. Separation was performed using a reversed­phase column (100 μm ID ×15 cm, Reprosil-Pur 120 C18­AQ, 1.9 μm, Dr. Maisch). Mobile phases were H2O with 0.1% FA, 2% ACN (phase A) and 80% ACN, 0.1% FA (phase B). Separation of sample was executed with a 120 min gradient at 300 nL/min flow rate. Gradient B: 2­5% for 2 min, 5­22% for 88 min, 22­45% for 26 min, 45­95% for 2 min, 95% for 2 min.

Data dependent acquisition (DDA) was performed in profile and positive mode with Orbitrap analyzer at a resolution of 120,000 (@200 m/z) and m/z range of 350­1600 for MS1; For MS2, the resolution was set to 15,000 with a dynamic first mass. The automatic gain control (AGC) target for MS1 was set to 3E6 with max IT 50 ms, and 1E5 for MS2 with max IT 110 ms. The top 20 most intense ions were fragmented by HCD with normalized collision energy (NCE) of 27%, and isolation window of 1.2 m/z. The dynamic exclusion time window was 45 s, single charged peaks and peaks with charge exceeding 6 were excluded from the DDA procedure.

Vendor’s raw MS files were processed using Proteome Discoverer(PD) software (Version 2.4.0.305) and the built-in Sequest HT search engine. MS spectra lists were searched against their species­level UniProt FASTA databases (uniprot-Mus musculus-10090-2022-11.fasta), Carbamidomethyl [C] as a fixed modification, Oxidation (M) and Acetyl (Protein N­term) as variable modifications. Trypsin was used as proteases. A maximum of 2 missed cleavage(s) was allowed. The false discovery rate (FDR) was set to 0.01 for both PSM and peptide levels. Peptide identification was performed with an initial precursor mass deviation of up to 10 ppm and a fragment mass deviation of 0.02Da. Unique peptide and Razor peptide were used for protein quantification and total peptide amount for normalization. All the other parameters were reserved as default.

***Primers***

GAPDH-F: 5′-TGTTTCCTCGTCCCGTAGA-3′; and GAPDH-R: 5′-CAATCTCCACTTTGCCACTG-3′;

IL-6-F: 5′-TAGTCCTTCCTACCCCAATTTCC-3′; and IL-6-R: 5′-TTGGTCCTTAGCCACTCCTTC-3′;

TNFα-F: 5′-CCCTCACACTCAGATCATCTTCT-3′; and TNFα-R: 5′-GCTACGACGTGGGCTACAG-3′;

Nrf2-F: 5′-TCTTGGAGTAAGTCGAGAAGTGT-3′; and Nrf2-R: 5′-GTTGAAACTGAGCGAAAAAGGC-3′;

HO-1-F: 5′-AAGCCGAGAATGCTGAGTTCA-3′; and HO-1-R: 5′-GCCGTGTAGATATGGTACAAGGA-3′;

ZO-1-F: 5′-AGGACACCAAAGCATGTGAG-3′; and ZO-1-R: 5′-GGCATTCCTGCTGGTTACA-3′;

Occludin-F: 5′-TGAAAGTCCACCTCCTTACAGA-3′; and Occludin-R: 5′-CCGGATAAAAAGAGTACGCTGG-3′;

Claudin-1-F: 5′-TGCCCCAGTGGAAGATTTACT-3′; and Claudin-1-R: 5′-CTTTGCGAAACGCAGGACAT-3′;

Hmgcs1-F: 5′-GGTGGGTCGGTGGCTATAAA-3′; and Hmgcs1-R: 5′-GCATGGTGAAAGAGCCAAAGG-3′;

**REFERENCE**

1. Chen K, Luan X, Liu Q, Wang J, Chang X, Snijders AM, et al. Drosophila Histone Demethylase KDM5 Regulates Social Behavior through Immune Control and Gut Microbiota Maintenance. Cell Host Microbe. 2019;25 4:537-52.e8. doi:10.1016/j.chom.2019.02.003.

2. Yang C, Xu Z, Deng Q, Huang Q, Wang X and Huang F. Beneficial effects of flaxseed polysaccharides on metabolic syndrome via gut microbiota in high-fat diet fed mice. Food Res Int. 2020;131:108994. doi:10.1016/j.foodres.2020.108994.

3. Sheng D, Zhao S, Gao L, Zheng H, Liu W, Hou J, et al. BabaoDan attenuates high-fat diet-induced non-alcoholic fatty liver disease via activation of AMPK signaling. Cell Biosci. 2019;9:77. doi:10.1186/s13578-019-0339-2.
